# Individual-specific functional connectivity predicts clinical symptoms severity in patients with post-traumatic stress disorder

**DOI:** 10.1186/s12888-026-07969-3

**Published:** 2026-03-12

**Authors:** Minhua Yu, Bo Rao, Wenyan Zhao, Feng Xiao, Huan Li, Jinying Zhao, Yuliang Zhou, Shi Cheng, Zhipeng Xu, Haibo Xu

**Affiliations:** 1 Department of Radiology, Zhongnan Hospital of Wuhan University, Wuchang District, Wuhan City, Hubei Province 430071 China; 2 Department of Neuropsychology, Zhongnan Hospital of Wuhan University, Wuhan, Hubei China; 3 Department of Neurology, Tangdu Hospital, Air Force Medical University, Xi ’an City, Shanxi 710000 China

**Keywords:** Post-traumatic stress disorder, Resting-state functional MRI, Networks, Individualization, Symptom prediction

## Abstract

**Background:**

Despite significant group-level findings on functional connectivity (FC) alterations in patients with Post-Traumatic Stress Disorder (PTSD), previous studies have failed to establish reliable neuroimaging biomarkers for diagnosis or symptom prediction. This exploratory study aims to investigate the potential predictive value of resting-state brain network FC at both individual-specific and group levels for clinical symptoms in PTSD patients, and to identify predictive FC biomarkers in specific functional networks.

**Methods:**

45 PTSD patients diagnosed according to Diagnostic and Statistical Manual of Mental Disorders (Fifth Edition) criteria (12 males/33 females, aged 28.64 ± 6.87 years) were enrolled. All participants underwent high-resolution T1WI and resting-state functional MRI sequence acquisitions using a 5.0 T MRI system. FC matrices were generated based on both individual-specific parcellation and group-level atlases. A support vector machine for regression model combined with leave-one-out cross-validation was applied to evaluate the predictive performance of FC for the Posttraumatic Stress Disorder Checklist-5 (PCL-5) scores in PTSD patients. Key functional connections identified as predictors of symptom severity were analyzed, and each individual-specific subregion was classified based on its membership within one of seven major brain networks. The large-scale brain network properties significant for predicting PTSD symptoms were further explored.

**Results:**

Functional connectivity derived from individual-specific parcellation significantly predicted PCL-5 scores in PTSD patients (*r* = 0.5275, *p* = 0.0010), whereas the association at the group-level was significantly reduced (*r* = -0.0360, *p* = 0.3710). Further analysis revealed that the key predictive functional connections primarily involved between-network connectivity and with dorsal attention network (DAN) contributed most significantly to the prediction of symptom severity. Interestingly, FC between networks was overall positively correlated with higher PCL scores (*r* = 0.5265, *p* = 0.0002).

**Conclusions:**

Individual-specific FC analysis demonstrated preliminary evidence of higher accuracy and relevance in predicting clinical symptoms of PTSD, with DAN contributing most significantly to the prediction. The predictive effect was primarily linked to bidirectional alterations in between-network connectivity, with a net positive correlation observed on average. These findings provide evidence for the utility of using individualized brain region homologies in predicting symptom severity within PTSD.

**Clinical trial number:**

Not applicable.

**Supplementary Information:**

The online version contains supplementary material available at 10.1186/s12888-026-07969-3.

## Introduction

Post-traumatic stress disorder (PTSD) is a psychiatric condition triggered by traumatic events, primarily characterized by intrusive memories, avoidance behaviors, hypervigilance, and negative alterations in emotions and cognition [1]. Previous studies have demonstrated that the development of PTSD is associated with widespread structural and functional abnormalities in the brain. Research has shown that PTSD patients exhibit reduced gray matter volume in brain regions including the prefrontal cortex (PFC), hippocampus [2], anterior cingulate cortex (ACC) [3], amygdala [4], and insula [5]. The classic functional mechanism involves PFC hypoactivity, which impairs its inhibitory control over the amygdala’s fear response, leading to PTSD-related fear reactions, with the hippocampus playing a coordinating role [6, 7]. Research utilizing connectome gradient analysis integrated with graph theory methods has revealed multi-level disruptions in the hierarchical organization of brain networks in patients with PTSD, suggesting that the impairment of cerebral hierarchical architecture may underlie clinical symptoms such as hypervigilance and dissociation in affected individuals [8]. Whole-brain connectome analysis revealed large-scale functional connectivity (FC) dysregulation in PTSD patients, primarily characterized by widespread weakening of connections within and between higher-order cortical networks, such as the default mode network (DMN) and central executive network (CEN), alongside enhanced connectivity from emotional and arousal systems (such as the thalamus and limbic regions) to sensory and default mode areas [9]. A meta-analysis has indicated that hypoconnectivity within the DMN and between the affective network (AN) and DMN specifically relates to traumatic experience, while hyperconnectivity linking the AN and somatomotor network (SMN) as well as the DMN and SMN was specifically associated with PTSD [10]. Alterations in FC within large-scale brain networks have become a key perspective for understanding the pathophysiology of PTSD.

Contemporary neuroimaging methodologies predominantly rely on group-level or population-based analytical approaches. These analytical approaches demonstrate efficacy in identifying significant neuroimaging-clinical correlations and elucidating generalized pathophysiological profiles, yet they frequently exhibit limited capacity to precisely delineate subject-specific neurobiological variability. This limitation significantly hinders their clinical utility in personalized diagnosis and therapeutic management. Conversely, individual-specific analytical frameworks explicitly incorporate intrinsic biological differences across subjects, offering enhanced diagnostic accuracy while simultaneously improving both symptom evaluation and prognostic prediction [11–13]. Recent findings have shown that using individual-specific FC has improved the predictive power of features that distinguish between responses to antidepressants and placebos in major depressive disorder [14]. These identified predictive biomarkers provided novel insights into the neuropathological mechanisms underlying treatment responses. A small-scale study by Brennan et al. reported that traditional group-level brain atlases did not identify resting-state FC biomarkers associated with symptom dimensions or severity in obsessive-compulsive disorder (OCD), whereas individual-specific FC analysis offered preliminary network-level insights related to symptom severity [15]. These findings should be interpreted cautiously due to the limited sample size.

Current approaches to individual-specific FC analysis often incorporate several strategies, such as data-driven parcellation methods that identify functional regions at the single-subject level, the generation of subject-specific functional connectomes, and the use of predictive modeling (e.g., support vector regression) trained on individual-level FC features. Furthermore, incorporating multimodal neuroimaging data can refine these individualized analyses by providing complementary constraints on brain organization at the subject level. Wang et al. introduced a novel individual-specific parcellation method to identify homologous functional brain regions [16], which accounts for inter-individual variability in FC. This individualized cortical functional network parcellation method utilizes an iterative optimization strategy that combines population atlas guidance with adaptive adjustment of individual fMRI signals. It achieves high within-subject reproducibility and effectively captures functional differences across individuals, particularly in higher-order cortical regions. This approach has been validated across diverse populations, including healthy individuals at 3T field strength [17], and patients with neuropsychiatric disorders such as schizophrenia [18]and Alzheimer’s disease [19]. However, its application to PTSD research remains unexplored.

In our initial validation using a 5T ultra-high-field MRI setup, we demonstrated the robust spatial and functional consistency of this individual-specific analytical framework in mapping individual brain networks [20]. Despite significant group-level findings on FC alterations in PTSD, previous studies have failed to establish reliable neuroimaging biomarkers for diagnosis or symptom prediction. To deepen our understanding of the neural mechanisms underlying PTSD and to elucidate how FC changes relate to clinical symptoms, this study employs an individualized parcellation strategy at 5T ultra-high field strength to examine both individual-specific and group-level resting-state FC patterns. Given the exploratory characteristic of this study, we conducted a whole-brain analysis with the following specific aims: (1) Compare the predictive value of individual-specific versus group-level FC patterns for PTSD symptoms; and (2) Identify candidate individual-specific FC patterns that may serve as potential biomarkers for further validation.

## Materials and methods

### Participants

This study was approved by the Ethics Committee of Zhongnan Hospital of Wuhan University, and all participants provided written informed consent. Participants were recruited primarily from the outpatient clinic of the Neuropsychology Department at Zhongnan Hospital. All participants received a formal diagnosis through a structured clinical assessment conducted by an experienced and licensed clinician specializing in neuropsychology, in strict accordance with the Diagnostic and Statistical Manual of Mental Disorders, Fifth Edition (DSM-5) criteria [21], which was issued by the American Psychiatric Association in May 2013. Fifty-three patients with PTSD were initially included and underwent MRI scanning. Following image quality control procedures (including head motion assessment), forty-five PTSD patients were ultimately enrolled in the study. To ensure a homogeneous and medication-naïve sample, all enrolled PTSD patients were carefully screened to confirm that they were not taking any psychotropic medications. Regarding psychiatric comorbidities, 24 participants presented with comorbid anxiety symptoms and 16 exhibited comorbid depressive symptoms. It is important to note that while these comorbid affective symptoms were common, the diagnosis of PTSD was established as the primary condition, and all participants met the full DSM-5 criteria for PTSD prior to study inclusion. Participants were assessed for PTSD symptoms using the Posttraumatic Stress Disorder Checklist-5 (PCL-5) [22–24]. The PCL-5 is a self-report instrument developed to screen for probable PTSD and evaluate the severity of its symptoms. Comprising 20 items, each rated on a 5-point scale from 0 (“not at all”) to 4 (“extremely”), the measure yields a total symptom severity score ranging from 0 to 80, obtained by summing responses across all items. It is important to note that the PTSD diagnostic assessment was performed independently of and prior to the administration of the PCL-5. The clinicians responsible for establishing the DSM-5 diagnoses were blinded to the participants’ subsequent PCL-5 scores, ensuring that the diagnostic classification was not influenced by the self-report symptom severity measure. Respondents rated their symptom severity over the past month, and the recommended cutoff score for PTSD screening was set at ≥ 33 points based on previous studies [25, 26]. Based on these criteria, we established the following inclusion and exclusion criteria for participants in this study.

Inclusion criteria for the PTSD patients: (1) aged 18–65 years and right-handed; (2) history of experiencing or witnessing severe traumatic events, meeting DSM-5 diagnostic criteria for PTSD; (3) PCL-5 score ≥ 33; (4) ability to tolerate MRI examination; (5) no history of central nervous system diseases including traumatic brain injury, brain tumors, stroke, or epilepsy; (6) no prior diagnosis of psychotic disorders. Exclusion criteria: (1) failure to meet the DSM-5 diagnostic criteria for PTSD; (2) presence of MRI contraindications or inability to tolerate MRI examination (e.g., claustrophobia, inability to cooperate with the procedure).

### MRI acquisition and quality control

In this study, all participants underwent brain scanning using the 5.0 T MRI system ((uMR Jupiter, United Imaging Healthcare, Shanghai, China). The T1-weighted structural images were acquired with a three-dimensional fast gradient echo sequence (T1 GRE-FSP 3D) with the following parameters: repetition time (TR) = 9.1 ms, echo time (TE) = 3.2 ms, slice thickness = 0.7 mm, flip angle (FA) = 9°, acquisition matrix = 312 × 352, field of view (FOV) = 220 mm × 248 mm, total slices = 240 (sagittal orientation), and voxel size = 0.7 × 0.7 × 0.7 mm³. Functional magnetic resonance images were acquired using a gradient echo-planar imaging (EPI) sequence with the following parameters: TR/TE = 1600 ms/28.5 ms, time points = 240, FOV = 211 mm × 211 mm, FA = 60°, acquisition matrix = 132 × 132, slice thickness = 1.6 mm, number of slices = 85, and voxel size = 1.6 × 1.6 × 1.6 mm³. The resting-state fMRI acquisition duration (240 time points × 1.6 s TR = 6.4 min) was determined to balance the requirements for high spatial resolution (1.6 mm isotropic) and whole-brain coverage at 5.0 T, while minimizing participant discomfort and motion-related artifacts, a critical consideration for clinical populations. The enhanced signal characteristics at ultra-high field strength support the acquisition of stable connectivity estimates within this timeframe, particularly when combined with individualized parcellation methods designed to improve sensitivity to subject-specific signals [16, 20]. Meanwhile, given that the functional sequence was configured for high-resolution isotropic imaging at 1.6 × 1.6 × 1.6 mm³, and considering potential geometric distortions caused by B0 field inhomogeneity inherent to ultrahigh-field MRI thin-slice blood oxygenation level dependent (BOLD) sequences, field maps were simultaneously acquired for subsequent correction. The field map parameters were configured as follows: TR = 10.6 ms, number of echoes = 2, TE1/TE2 = 4.92/7.56 ms, FOV = 211 mm × 211 mm, FA = 8°, acquisition matrix = 132 × 132, slice thickness = 1.6 mm, number of slices = 85, and voxel size = 1.6 × 1.6 × 1.6 mm³. To ensure both subject comfort and optimal image quality, the participants were instructed to relax their bodies, lie comfortably with their eyes closed, remain awake, and minimize cognitive activity during scanning. Additionally, foam padding was employed to stabilize head position and reduce motion artifacts, while earplugs were provided to attenuate scanner noise. These measures collectively guaranteed successful MRI acquisition and maintained high image quality standards.

To ensure the quality of MRI data, we implemented a two-stage quality control system. During MRI sequence acquisition, we monitored scan quality in real-time to address any issues such as noticeable artifacts or excessive head motion. In cases where these problems were evident, repeat scans were requested, or in severe cases, the participant’s data was excluded to maintain analytical accuracy. Additionally, for subtle motion assessment and correction, we employed framewise displacement (FD) metrics to control motion effects [27]. Participants with mean FD exceeding 0.5 mm were excluded to ensure data quality [28, 29].

### Neuroimaging data preprocessing

Resting-state fMRI data preprocessing was performed using the Data Processing & Analysis for Brain Imaging (Version 8.1) (DPABI V8.1) [30] and the Statistical Parametric Mapping toolbox (SPM12, https://www.fil.ion.ucl.ac.uk/spm), with the software running in the MATLAB 2020a environment. The processing workflow comprised the following steps: (1) removal of the first 10 time points; (2) slice timing correction; (3) realignment for head motion correction; (4) field map correction, a critical step in fMRI data preprocessing to address spatial distortions caused by magnetic field inhomogeneities. This part was conducted using the FMRI Expert Analysis Tool (FEAT) in FMRIB’s Software Library (FSL), with the field-map corrected images showing significant improvement in geometric distortion; (5) regression of covariates (including white matter, cerebrospinal fluid, global signal, and Friston’s 24 parameter model); (6) bandpass filtering (0.01–0.08 Hz).

Structural MRI data were processed using the FreeSurfer 6.0.0 software package in a Linux environment. The workflow involved boundary-based registration to align the structural and functional images. The preprocessed fMRI data of each subject were first registered to the FreeSurfer surface template *fsaverage6*, which consists of 40,962 vertices per hemisphere. Subsequently, the cortical surface fMRI data were smoothed using a 6-mm full-width-at-half-maximum (FWHM) Gaussian kernel. Finally, the smoothed data were down-sampled to the *fsaverage4* template (2562 vertices per hemisphere) using the *mri_surf2surf* command from the FreeSurfer toolkit for further analysis.

### Identification of individual-specific functional regions of interest (ROIs) and FC estimation

Following preprocessing of fMRI data, individual-specific functional networks were parcellated, homologous ROIs were identified, and ROI-ROI FCs were calculated using the Homologous Functional Regions Across Individuals toolbox (HFR_ai, http://nmr.mgh.harvard.edu/bid/DownLoad.html). HFR_ai was executed in the FreeSurfer environment, following a stepwise analytical pipeline based on previously reported methods in the literature [16, 18, 19].

Step 1: An iterative parcellation approach was developed to meticulously delineate 18 functional networks in individual subjects [16], based on the functional brain atlas reported by Yeo et al. in 2011 [31]. The original Yeo atlas comprised 17 brain networks further subdivided into 114 discrete brain ROIs. Through a sensorimotor hand motor task, the sensorimotor hand area was specifically identified, resulting in a final atlas comprising 116 ROIs and forming a group-level functional atlas of 18 cortical networks. Using a population-level functional network atlas constructed from 1,000 healthy subjects as the initial reference, the group atlas was projected into individual space, and the mean BOLD signal of each network was established as the initial reference signal. Subsequently, network affiliation assignment was performed by calculating the correlation between individual vertex signals and the reference signal, with a reliability threshold (set to the default value of 3 in this study, based on previously validated parameters in studies using this individual parcellation method) applied to filter reliable vertices for generating core signals [16, 17]. During the iterative process, the fusion weights between core signals and reference signals were dynamically adjusted (based on individual variability and signal-to-noise ratio distribution), progressively reducing the influence of group-level data. The optimization terminated when the network overlap rate reached a convergence threshold or after completing a preset number of iterations (default set to 10 in this study), yielding high-precision, individually driven functional network parcellation results.

Step 2: The identification of homologous functional networks at the individual level [17]. The HFR_ai software, in conjunction with the clustering algorithm from FreeSurfer (using the mri_surf_cluster command tool), was utilized to parcellate each cortical network from Step 1 into discrete “patches” for each individual subject. To minimize noise and matching rate effects, the cortical networks were smoothed with a 1 mm Gaussian kernel to reduce noise interference. Subsequently, individual discrete functional “patches” were matched to the 116 ROIs of the group atlas as follows: (1) If a patch at the individual level overlapped with a single ROI in the group-level template, it was labeled as the same ROI as the atlas; (2) If an individual-level patch overlapped (more than 20 vertices) with multiple ROIs in the template, it was divided into several smaller ROIs. Specifically, vertices overlapping between the individual-level patch and template ROIs were identified to form the centers of several smaller ROIs. The remaining vertices in the original patch were then assigned to the nearest ROI based on their geodesic distance along the cortical surface. (3) If an individual-level patch did not overlap with any template ROI and the shortest distance between the patch and an ROI fell within a specified threshold, the patch was assigned to its nearest ROI; otherwise, it was labeled as “unidentifiable”. This threshold was defined as the average distance between any two vertices within the nearest ROI in the template.

Step 3: FC between homologous brain ROIs at the individual level was computed. The FC of individual brain regions was represented by the signal correlation strength between functionally consistent ROIs obtained through individual parcellation. The mean BOLD signal across all vertices within each ROI was extracted as the representative BOLD signal for that ROI. Pearson correlation coefficients were then calculated to estimate pairwise FC between ROIs, followed by Fisher’s z-transformation to convert correlation coefficients into z-values. This process yielded an FC matrix based on individual-level homologous brain ROIs, while simultaneously generated a corresponding group-level atlas-based FC matrix using the same ROI definitions.

### Prediction of individual PTSD symptoms using individual-specific/atlas functional connectivity

**Fig. 1:**
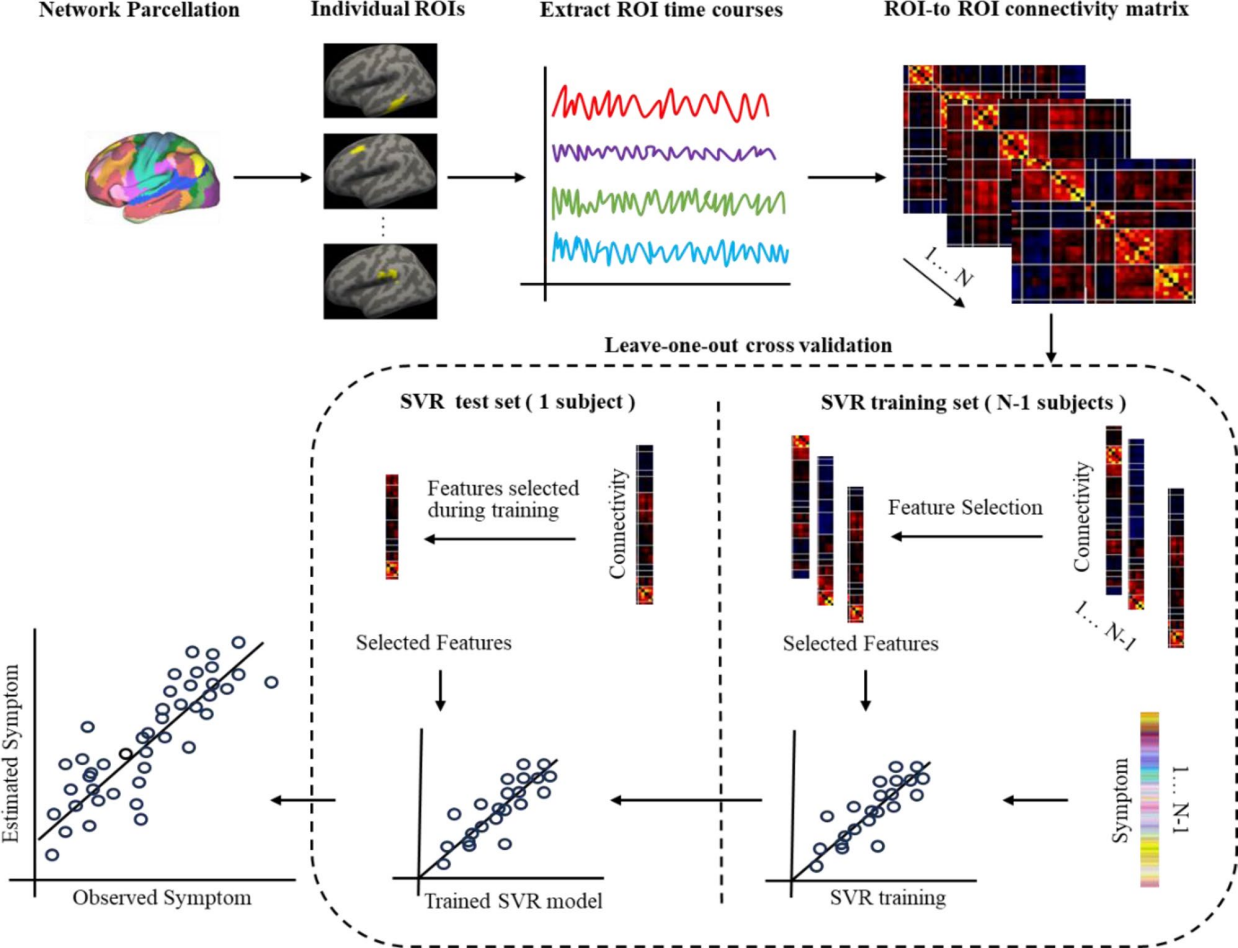
The SVR workflow for predicting PCL scale scores using FC between individual-specific ROIs (the dashed box indicates the LOOCV procedure) was implemented as follows. Based on individualized parcellation, we identified 82 homologous brain ROIs across all participants. Using the FC features from these parcellated ROIs, we trained an SVR model to estimate the PCL-5 scores for each patient. To reduce data dimensionality, only the FC subset showing significant correlations with symptom scores in the training dataset (N-1 subjects; 44 cases in this study) was selected as features for SVR model training. The resulting model was then applied to the remaining single test subject to predict their scale score. This procedure was repeated N times to predict scores for all participants. Finally, we evaluated the correlation between predicted and actual PCL-5 scale scores. ROI: region of interest. SVR: support vector regression

Based on individualized parcellation-derived homologous brain ROIs and the extracted network atlas, we successfully identified 82 ROIs across 18 brain networks. The resting-state FC between individual-specific and the corresponding atlas-level ROIs were calculated. Subsequently, these FC matrices were utilized to predict patients’ PCL-5 scale scores, and the predictive capability of the individualized parcellation method for PTSD symptoms was evaluated through correlation analysis (the workflow is illustrated in the Fig. 1 ).

To achieve this, we first calculated resting-state FC between individual-specific ROIs to predict patients’ PCL-5 scale scores. We employed a support vector machine for regression (SVR) model to estimate the scale scores for each PTSD patient, specifically using the SVR model from the MATLAB Statistics and Machine Learning Toolbox (with a Gaussian kernel function). Given the relatively small sample size of this study, we applied leave-one-out cross-validation (LOOCV) to ensure the accuracy and reliability of the evaluation. In each LOOCV iteration, the FC data of one subject were held out as the test set, while the remaining N-1 (44 cases in this study) samples were used as the training set to build the model and predict the scale score of the left-out subject. This process was repeated for each subject, ultimately generating predicted PCL-5 scores for all participants. The performance of the predictive model was assessed by computing the correlation between the predicted and the actual measured PCL-5 scale scores. To rule out spurious correlations, a non-parametric permutation test was performed: observed symptom scores were randomly reassigned across participants, and the symptom estimation procedure was repeated for 1,000 permutations. The permutation p-value was determined by calculating the proportion of permutations in which the estimated-observed correlation coefficient exceeded that obtained from the original data. Given the relatively small sample size in our study and the absence of statistically significant correlations between factors such as age, gender, head motion, and PCL-5 scale scores (see Supplementary Materials), we intentionally refrained from routinely incorporating these covariates into the primary predictive model to mitigate the risk of overfitting. Additional technical details are provided in the Supplementary Materials.

In each iteration of the LOOCV procedure, feature selection, model training, and testing were performed. For this study, the input features consisted of FC values between all ROI pairs (82 × 82). We then reduced feature dimensionality by computing the correlation between symptom scores and FC values within the training set, retaining only those FC features that showed a significant association with the scale scores (significance threshold: *p* < 0.005; see Supplementary Materials) for training the SVR model. Finally, the selected features from the test data (i.e., the left-out subject in each LOOCV iteration) were input into the trained model to predict the corresponding subject’s PCL-5 score.

Using the identical analytical approach, we also evaluated the predictive efficacy of atlas-based ROIs FC for PTSD clinical symptoms and conducted a comparative analysis with the predictive performance obtained at the individual-specific ROIs.

### Contribution of each ROI and network to clinical symptom prediction

Different FC features exhibited varying contributions to the predictive outcomes within this regression framework. Notably, certain inter-regional connections demonstrated greater predictive influence, as quantified through the model’s weight metrics. During each LOOCV iteration, the trained SVR model assigned specific weights to individual features. By aggregating weights across all LOOCV cycles, we identified which connection played pivotal roles in predicting symptom severity, while connections consistently excluded by the model were deemed non-contributory. To evaluate the predictive contributions of different ROIs, we calculated the summed weights of all functional connections associated with each specific ROI. Any ROI that showed no association with predictive FCs was assigned a zero contribution value.

To further evaluate the predictive value of large-scale brain network FC for PTSD symptoms, and to analyze both within-network and between-network weights associated with each functional network, we clustered the individually parcellated ROIs according to their membership in seven major large-scale brain networks. Based on whether the parcellated ROIs comprising predictive FC pairs belonged to the same network, functional connections were classified as either within-network connections (linking regions within the same network) or between-network connections (linking regions across different networks). By aggregating the corresponding weights of all functional ROIs within each large-scale network, we derived both within-network and between-network weights for each major large-scale brain network. This approach enabled identification of which large-scale brain networks demonstrated particularly high predictive value for PTSD symptoms, thereby facilitating the exploration of potential individual-specific functional network biomarkers for PTSD at the neurobiological level.

### Statistical analyses

Statistical analyses of clinical baseline characteristics were performed using SPSS 23.0 software (SPSS, Inc., Chicago, IL, USA). Continuous variables were compared using independent two-sample t-tests, with statistical significance defined as *p* < 0.05. Correlation analyses were performed using Pearson’s correlation tests, also with a significance threshold of *p* < 0.05. Prior to t-tests, normality was confirmed using the Shapiro - Wilk test (*p* > 0.05), and homogeneity of variances was assessed with the F‑test (*p* > 0.05). For Pearson correlations, linearity between variables was visually verified through scatterplots.

### Visualization

FC features between parcellated ROIs were visualized using Circos (http://circos.ca/) to create intuitive graphical representations.

## Results

### Demographic characteristics

All demographic and clinically relevant characteristics of the forty-five PTSD participants are presented in the Table 1.

**Table 1:** Demographic characteristics of all participants

	PTSD patients (*n* = 45)
Age, years, mean ± SD	28.64 ± 6.87
Gender, female, n (%)	33 (73.33%)
Education, years, mean ± SD	14.89 ± 2.57
Hypertension, n (%)	0 (0%)
Hyperlipidemia, n (%)	0 (0%)
Diabetes, n (%)	0 (0%)
PTSD duration, months, mean ± SD	4.00 ± 1.73
PCL-5 scores, mean ± SD	48.11 ± 10.15
Traumatic events	
Domestic violence	17 (37.78%)
Sudden death of loved one	13 (28.89%)
Divorce	9 (20.00%)
Other adverse social events	6 (13.33%)

Notes: PTSD: post-traumatic stress disorder; SD: standard deviation; PCL-5: Posttraumatic Stress Disorder Checklist-5

### Parcellated and identified homologous individual-specific ROIs

**Fig. 2:**
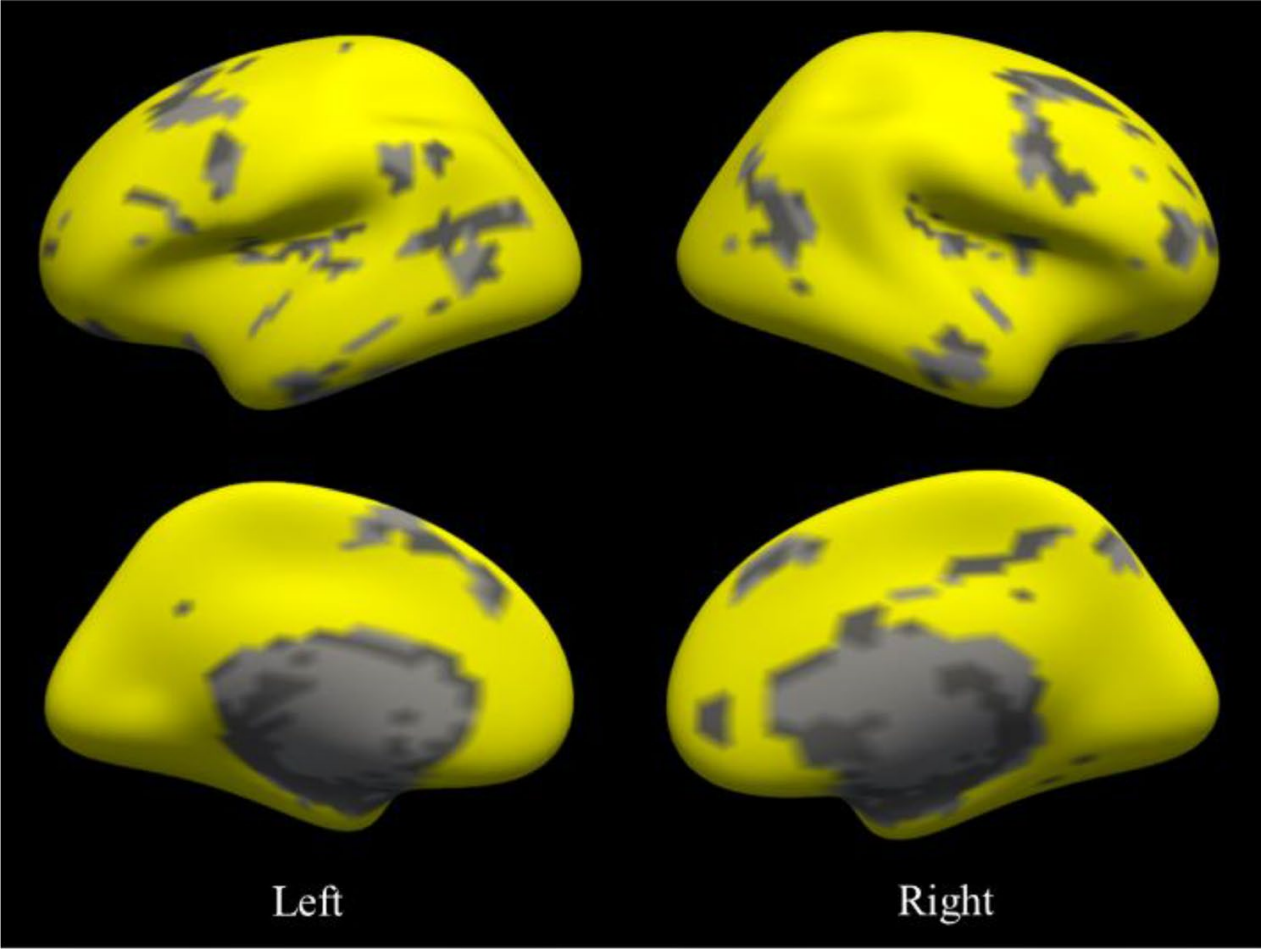
The cortical atlas of individualized ROIs from a representative participant. Yellow: identified/included individualized ROIs; Gray: unidentified /excluded ROIs

A total of 82 ROIs were identified across 18 brain networks for each participant, accounting for approximately 70.69% of the total candidate ROIs. The remaining 34 ROIs were categorized as “unidentified”. The aggregated set of individualized ROIs covered an average of 4155.51 ± 147.94 vertices across all participants, corresponding to a coverage rate of 81.10% ± 2.89%. The cortical projection atlas of the individualized ROIs from a representative participant is presented in Fig. 2.

### Individual-specific functional connectome tracks PTSD symptoms

**Fig. 3:**
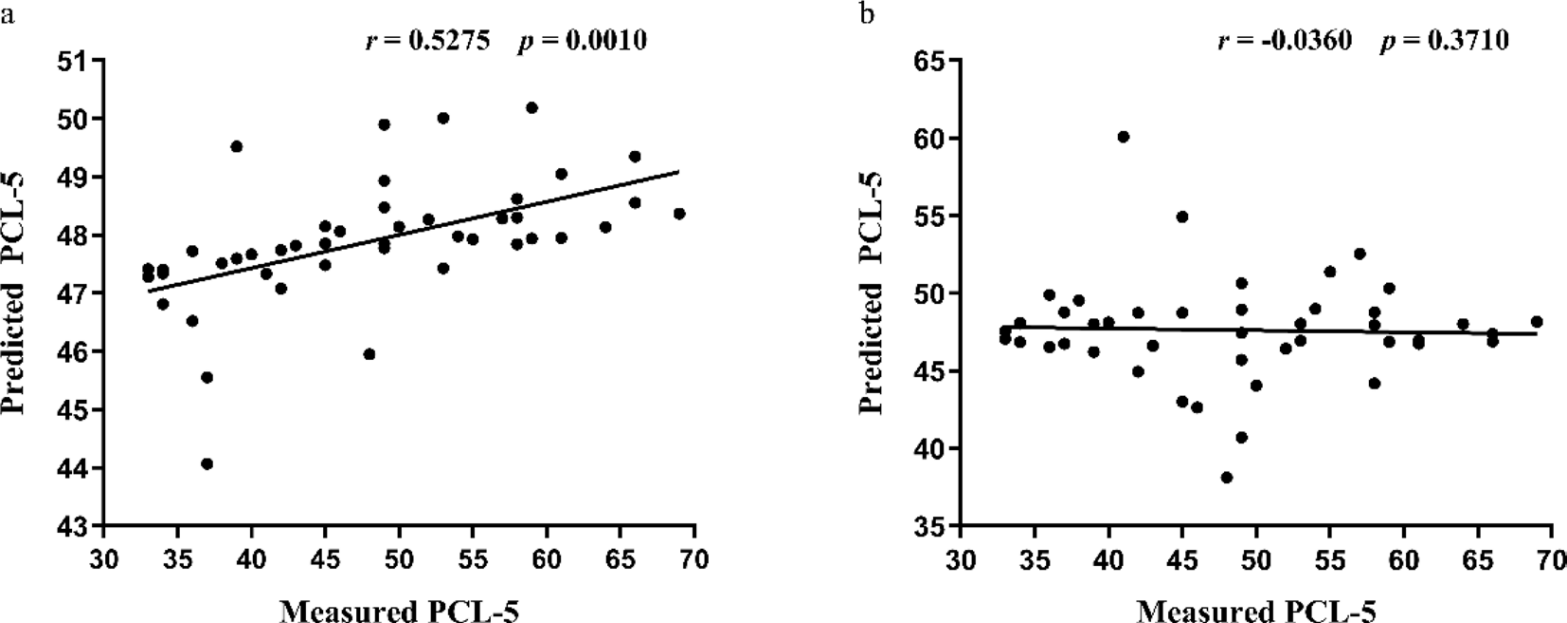
FC between individual-specific and atlas-based ROIs to predict PCL-5 scores in PTSD patients. (**a**) FC based on individual-specific ROIs effectively predicted PCL-5 scores (showing a significant positive correlation with the actual measured scores); (**b**) FC derived from the Yeo’s atlas-based ROIs failed to predict PCL-5 scores (no significant correlation with the actual measured scores). Pearson correlation analysis was applied, with statistical significance set at *p* < 0.05. Correlation significance was determined by using 1000 permutations

To investigate whether individually specified FC tracks with the PTSD symptoms, we trained SVR models to assess the predictive effects of individual-specific and atlas-based ROIs FC on PCL-5 scale scores in patients. The results demonstrated a significant correlation between the scale scores predicted by FC among individual-specific ROIs and the actual measured scale scores (r(43) = 0.5275, *p* = 0.0010, permutation test, Fig. 3a), whereas no significant correlation was observed between the predicted scores based on FC among ROIs defined by the Yeo’s group level atlas and the actual measured scale scores (r(43) = -0.0360, *p* = 0.3710, permutation test, Fig. 3b).

**Fig. 4:**
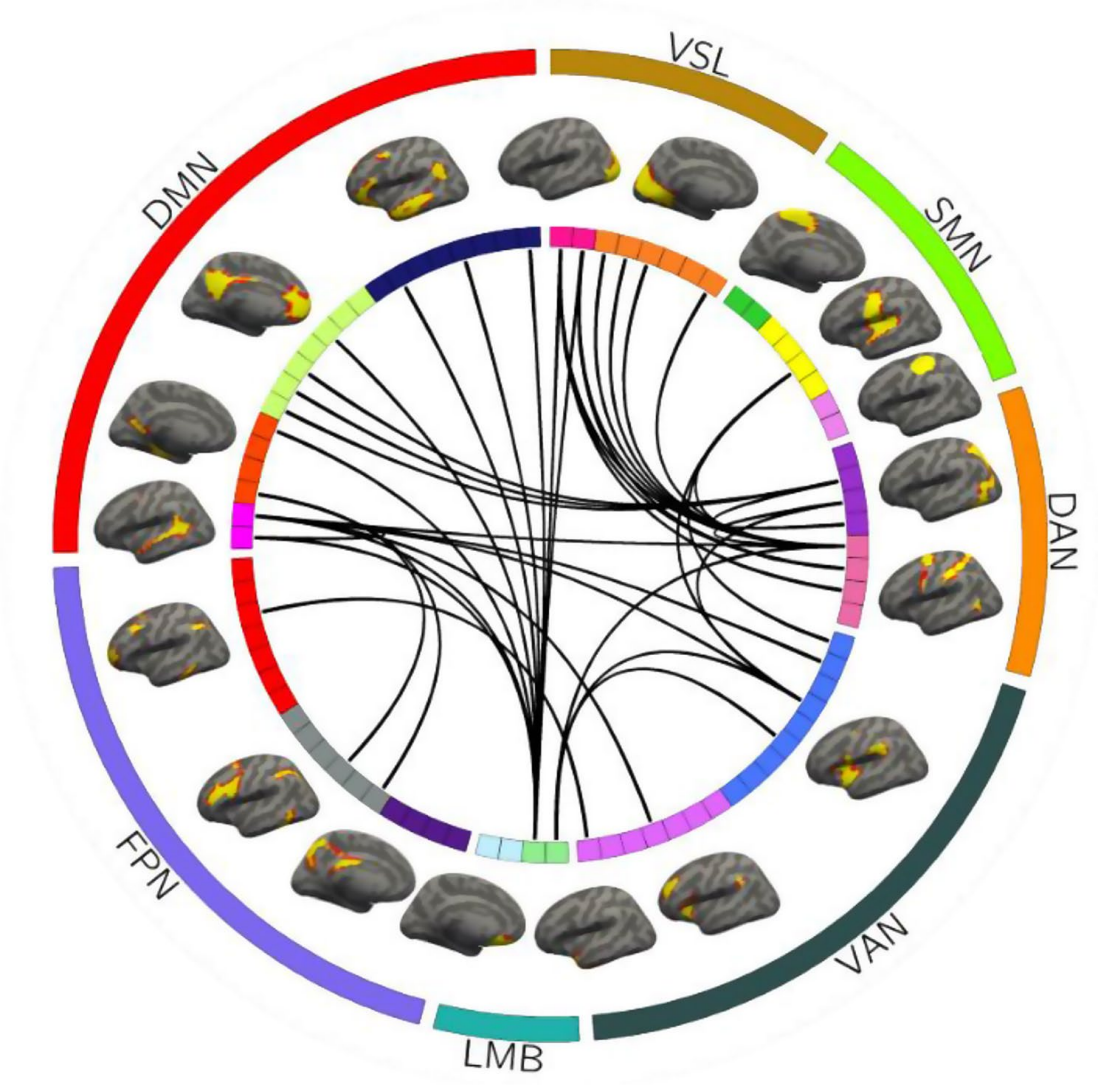
82 ROIs derived from the 18 networks are presented by the colored rectangles under the corresponding brain networks. The 38 predictive functional connections of PTSD scale scores identified by the SVR model and their corresponding functional ROIs are specified by the black lines. VSL: Visual network; SMN: Somatomotor network; DAN: Dorsal attention network; VAN: Ventral attention network; LMB: Limbic network; FPN: Frontoparietal network; DMN: Default mode network

At the individual-specific level, the SVR model identified 38 functional connections capable of predicting PTSD scale scores, with their corresponding functional ROIs and connections illustrated in Fig. 4.

**Fig. 5:**
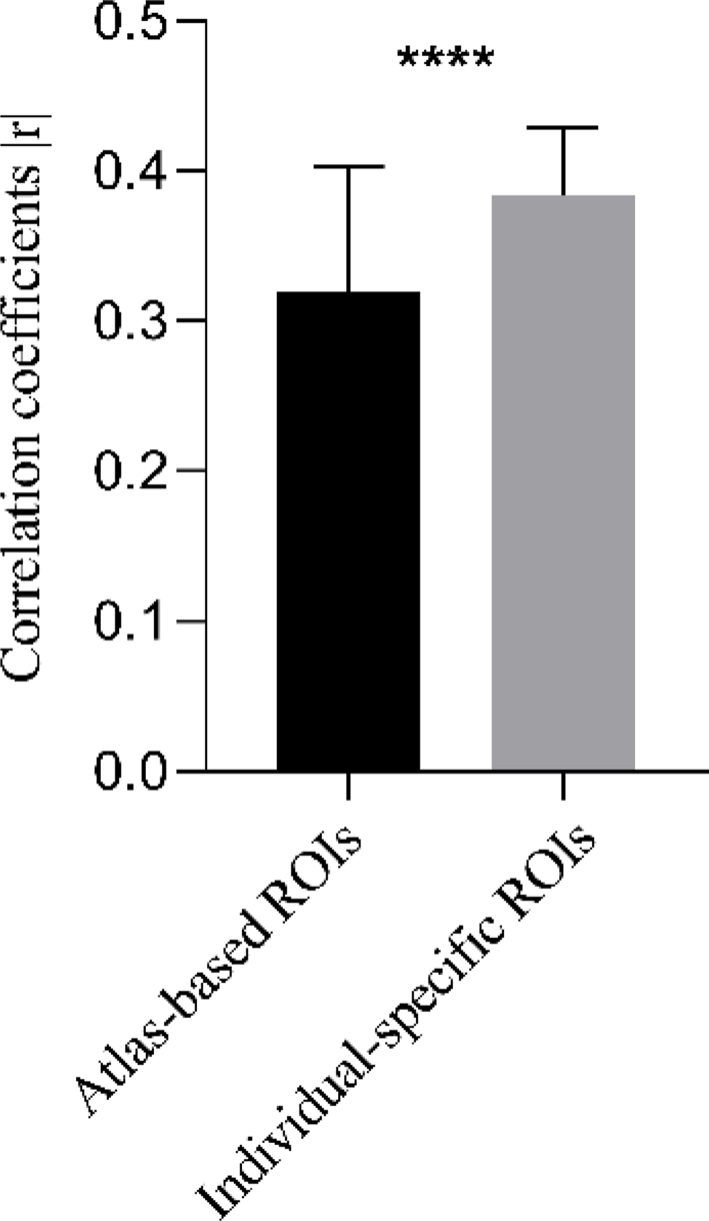
Differences in correlations of predictive FC and scale scores were compared between individual-specific and atlas-based approaches. Significantly reduced absolute r-values were observed at the atlas-based level (η² (37) = 0.5176, *p* < 0.0001, paired between-group t-tests)

To further validate whether the Yeo’s atlas-based ROIs reduced the correlation between FC features and PCL-5 scale scores, we redefined the predictive functional ROIs at the group atlas level. Specifically, we extracted the raw values of corresponding functional connections from both individual-specific and atlas-based matrices, then calculated their correlations with the actual PCL-5 scores at both group atlas and individual-specific levels. The absolute values of correlation coefficients (|r|) were compared between groups to evaluate whether the group atlas-level definitions weakened the connectivity-score correlations relative to individual-specific definitions. These results demonstrated that when predictive connections were defined at the group atlas level, their correlations with scale scores were significantly reduced (as shown in Fig. 5). This indicates that the association strength between atlas-defined functional connections and clinical scores was attenuated, thereby compromising the predictive power of these ROI-based functional connections.

### Contribution of network to PTSD symptom prediction

**Fig. 6:**
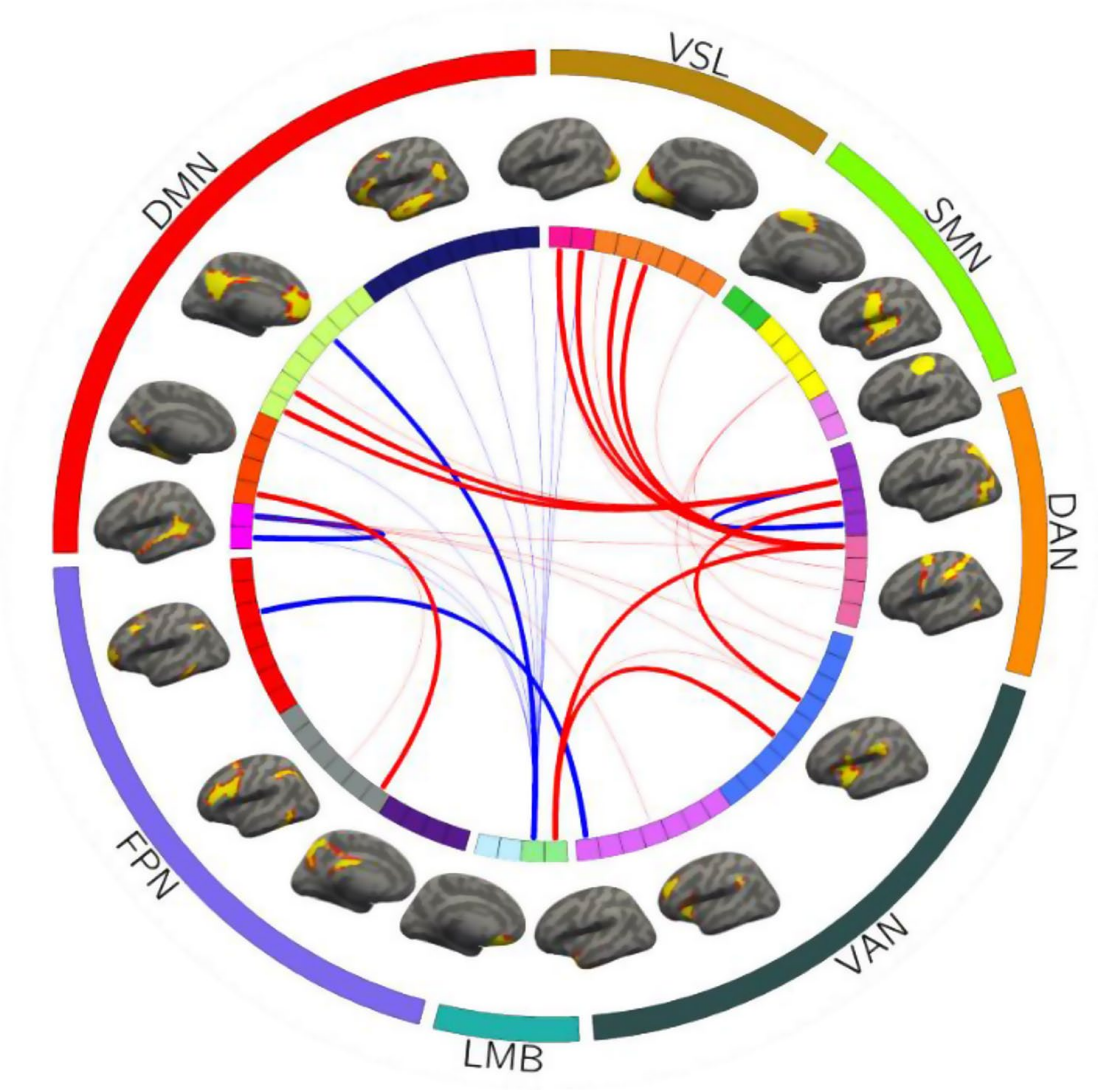
38 predictive functional connections were characterized by their network distributions and attributes. The top 14 connections ranked by predictive weight contribution are displayed with thickened lines, with connections positively correlated with scale scores marked in red and negatively correlated marked in blue. VSL: Visual network; SMN: Somatomotor network; DAN: Dorsal attention network; VAN: Ventral attention network; LMB: Limbic network; FPN: Frontoparietal network; DMN: Default mode network

**Fig. 7:**
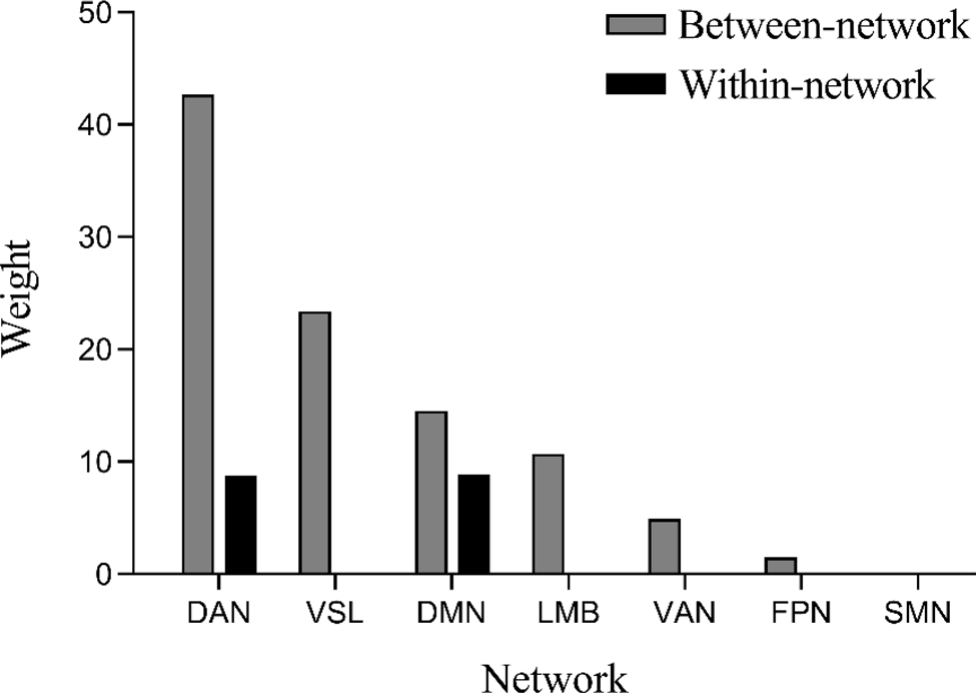
The predictive weights of within-network and between-network functional connections for PCL-5 score estimation were analyzed. Between-network connections primarily involved DAN, VSL, DMN, LMB and VAN, with DAN demonstrating the highest predictive weight, followed by VSL, DMN and LMB. Within-network connections were restricted to the DAN and DMN regions. DAN: Dorsal attention network; VSL: Visual network; DMN: Default mode network; LMB: Limbic network; VAN: Ventral attention network; FPN: Frontoparietal network; SMN: Somatomotor network

**Fig. 8:**
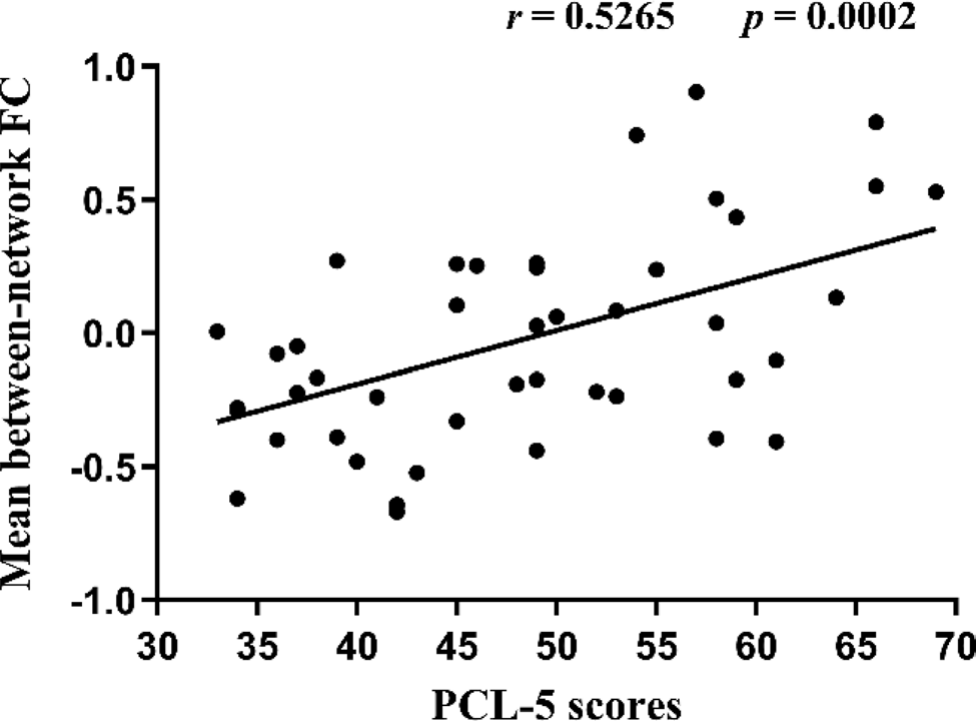
The correlation between averaged between-network FC and PCL-5 scores. The averaged between-network connections with predictive value showed significant positive correlation with PCL-5 scores, as determined by Pearson’s correlation analysis (*p* < 0.05)

The results of predictive FC properties demonstrated that 35 predictive functional connections were between-network connections, while only 3 were within-network connections. These between-network connections primarily involved the dorsal attention network (DAN), visual network (VSL), DMN, limbic network (LMB), and ventral attention network (VAN), with the highest weight attributed to the DAN, followed by the VSL, DMN, and LMB. The within-network connections involved brain networks of the DAN and DMN. Additionally, the prediction of PCL scale scores was associated with both strengthening and weakening of between-network connections, as well as weakening of within-network connections. These findings are illustrated in Figs. 6 and 7. To further investigate whether the prediction of PCL scale scores was linked to the strengthening or weakening of between-network connections, we averaged the original values of individual-specific between-network functional connections with predictive contributions for each subject and calculated the correlation between the mean connectivity values and scale scores. The results revealed a significant positive correlation between the mean between-network connectivity and scale scores (r(43) = 0.5265, *p* = 0.0002) (as shown in Fig. 8).

## Discussion

Neuroimaging holds promise for clarifying psychiatric mechanisms and predicting clinical features [32, 33], yet faces challenges such as modest effect sizes, limited replicability, and inter-individual variability-issues central to the present study. While group-level analyses in PTSD have revealed dysregulated cross-network connectivity and its association with symptoms [10, 34, 35], such approaches often fail to capture individual-specific patterns, particularly within highly variable brain regions [36]. This exploratory study employed 5T MRI combined with individualized cortical parcellation and machine learning to compare the predictive utility of individual-specific versus group-level resting-state FC for PTSD symptom severity. The primary findings revealed that individual-specific connectivity significantly predicted symptom severity, whereas atlas-based connectivity showed no predictive value. Predictive connections were primarily characterized by between-network interactions, notably involving DAN, DMN, VSL, VAN and LMB, with DAN contributing most substantially. The overall predictive effect was associated with bidirectional alterations in between-network connectivity, and demonstrated a net positive correlation with symptom severity.

The homologous brain regions delineated via individualized parcellation encapsulate subtle individual-level variations, thereby furnishing enhanced precision and richer informational content for diagnostic and evaluative processes in disease contexts [37]. Previous studies utilizing individualized parcellation and machine learning have consistently demonstrated superior predictive performance for clinical symptoms compared to group-level atlases across various psychiatric disorders. For instance, in OCD, individualized FC identified cortical-subcortical network biomarkers that were not detectable with group atlases and better predicted symptom severity and dimensionality [15]. Similarly, in schizophrenia, individual-specific connectivity markers tracked both dimensional and categorical psychotic features more accurately than group-level approaches [18]. In Alzheimer’s disease, individualized connectivity improved the prediction of cognitive symptoms regardless of apolipoprotein E (APOE) ε4 genotype [19]. This present study has also shown that individual-specific analytical methods outperform group-based atlas analysis in predicting PTSD symptoms. The consistency across disorders highlights the general advantage of individualized neuroimaging methods in capturing clinically relevant neural variability that is obscured in group-averaged data. Individual-specific analyses are designed to better capture structural and functional variations among different subjects, thereby providing an enhanced framework for addressing the inherent limitations of traditional group-level analytic approaches [38]. However, this is notable given the extensive literature documenting group-level FC alterations in PTSD. We posit that this result may be attributable to two interrelated factors. First, the specific group atlas used, while widely adopted, employs a relatively coarse parcellation (18 networks/116 ROIs). Larger ROIs may homogenize subtle, subject-specific FC variations that are clinically informative. It is plausible that a finer-grained group atlas (e.g., Glasser et al., 2016; Schaefer et al., 2018) with hundreds of parcels might better capture some of this detail and improve group-level prediction, a valuable direction for future research [39, 40]. Second and more fundamentally, the near-complete lack of predictive value at the group level underscores the significant challenge posed by inter-individual variability in functional neuroanatomy. In a clinically heterogeneous disorder like PTSD, the precise spatial location of symptom-relevant network nodes and connections may differ substantially across individuals.

Further analysis of individual-specific ROIs FC predictive of PTSD symptoms revealed that the majority of these FCs were between-network connections, primarily involving DAN, DMN, VSL, VAN and LMB. These findings imply that FC alterations in PTSD involve large-scale brain networks. Predictive connections are distributed across nearly the entire brain, reflecting the complexity of the disorder’s neural underpinnings. Previous studies have consistently identified widespread and intricate large-scale brain network FC abnormalities in PTSD patients, with altered within and between-network FC potentially linked to the onset and progression of PTSD [10, 41]. For instance, hypervigilance and reactivity symptoms are associated with heightened activation of the amygdala and dorsal anterior cingulate cortex, both key nodes of PFC [42]. Adolescent PTSD correlates with reduced intra-DMN connectivity and increased salience network (SN)-DMN connectivity [43]. Classical triple-network model studies have also confirmed within-network alteration and between-network dysregulation in PTSD [44, 45]. A recent study of Yale University employing connectome-based predictive modeling found that altered connectivity within and between SN, anterior DMN, and CEN at 1 month post-trauma could predict clinical symptoms at 14 months post-trauma [46]. Notably, in our study, DAN exhibited the highest predictive weight, suggesting its critical role in symptom prediction. The dominant contribution of DAN centrally involved in top-down attentional control, spatial orientation, and sensory selection-may reflect key pathophysiological processes in PTSD. While classical PTSD network models often emphasize the SN, DMN and CEN, our individualized findings highlight DAN as a potential pivotal hub in cross-network dysregulation. It is worth noting that the study of Yale University also indirectly suggested the involvement of dorsal attention pathways in predictive models [46]. Altered connectivity within the DAN-centered network may result in excessive allocation of attention to threat-related stimuli and impaired disengagement from emotional stimuli, which are core characteristics of hypervigilance and hyperarousal in PTSD [41]. VSL is also involved in the development of PTSD. PTSD patients exhibit abnormalities in both resting-state and task-based FC within VSL [46]. This suggests that their visual processing system, particularly pathways related to attention and emotion, may have altered sensitivity to trauma-related stimuli. Overall, our results extend these findings by demonstrating that individualized parcellation captures DAN-centered network interactions that are obscured in group-level analyses, thereby offering a more sensitive biomarker for symptom prediction.

Although LMB contributed to the prediction of PTSD symptoms in our model, its influence was less prominent than that of DAN or VSL. Moreover, our individualized cortical parcellation approach did not identify specific limbic subregions (such as the amygdala or hippocampal subdivisions) as significant standalone predictors. This may be attributed to several methodological considerations. First, our parcellation pipeline was optimized for cortical network identification and may have limited sensitivity to subcortical structures due to their higher anatomical variability and susceptibility to geometric distortion at 5T field strength, as observed in our earlier validation study [20]. Second, the predictive model primarily leveraged between-network connectivity, suggesting that PTSD symptom severity in this cohort may be more closely tied to cross-network communication than to intra-limbic circuitry. This finding does not undermine the well-established role of limbic structures in PTSD pathophysiology but highlights the importance of network-level dysregulation in mediating clinical symptoms. Future studies combining ultra-high-field MRI with subcortical segmentation techniques may help clarify the specific contributions of amygdala and hippocampal subfields to individualized prediction models in PTSD.

Our integrated analysis indicates that the predictive efficacy for PTSD symptoms primarily stems from bidirectional alterations in between-network connectivity, showing an overall positive association. This suggests that widespread network dysregulation exacerbates global symptom severity. This pattern of heightened connectivity may reflect a loss of network segregation or “dedifferentiation”, exemplified by the weakened anticorrelation between DAN and DMN, which could underlie the persistent self-referential or intrusive processing characteristic of PTSD [47]. The classic SN has been established as a core circuit associated with symptoms such as hypervigilance in PTSD [44, 48]. Although in our individualized model, its corresponding regions (primarily distributed across VAN and LMB) were not the highest-weighted predictors, they still participated in multiple cross-network interactions, particularly in connections between VAN and LMB. Notably, VAN demonstrated a clear predictive role in between-network connectivity, extending the conventional understanding of PTSD pathophysiology centered on SN. These findings suggest that individualized analytical methods can more comprehensively reveal brain network abnormalities underlying PTSD symptoms. Moving forward, differentiating distinct symptom clusters may further enhance the clinical utility of predictions and provide a basis for network-informed neuromodulation and targeted psychotherapy.

Our findings also revealed that reduced within-network FC within the DAN and DMN plays a significant role in predicting PTSD scale scores. Although only three within-network connections were identified, their high predictive weights indicate a clear relevance for symptom prediction. A meta-analysis demonstrated decreased FC within the DMN, which is centered on the medial PFC (mPFC) in PTSD patients [10]. Reduced functional activation of the mPFC is one of the classic circuit mechanisms in PTSD [49], and the observed reduction in intra-DMN FC effectively predicting clinical symptoms further supports its role in the disease pathology. Additionally, Evans et al. have revealed that PTSD symptoms are associated with diminished brain-behavior synchronization in the DAN, selectively impairing sustained attention and DAN engagement [50]. These findings collectively provide varying degrees of explanation and support for the predictive value of reduced intra-DAN and intra-DMN FC in PTSD symptomatology. What’s more, while such reductions are often viewed as reflecting pathological disruption of network integrity, they could also potentially represent adaptive, compensatory reorganization. In this framework, the brain might downregulate certain within-network connections to reallocate neural resources toward between-network interactions that become hyper-engaged in PTSD, as observed in our between-network connectivity findings. The current cross-sectional data cannot definitively distinguish between pathological dysfunction and compensatory adaptation. However, the predictive utility of these within-network reductions for symptom severity underscores their functional relevance. Future longitudinal studies assessing network changes pre- and post-treatment, or in individuals with varying levels of resilience, could help elucidate whether these connectivity patterns are maladaptive or represent attempted compensation.

In this cross-sectional study, individualized FC patterns were significantly associated with concurrently assessed PTSD symptom severity, suggesting their potential utility as neuroimaging markers for symptom characterization. However, the cross-sectional design limits causal or temporal inferences regarding the relationship between neural connectivity and symptom progression. The term “prediction” herein refers specifically to the identification of associations with current symptom severity, rather than forecasting future clinical trajectories. Future longitudinal studies are needed to determine whether such connectivity features can predict long-term symptom courses, treatment response, or clinical outcomes, thereby advancing the development of neurobiologically informed personalized interventions for PTSD.

This study has several limitations. First, although PTSD diagnoses were made by experienced clinicians using structured interviews per DSM-5 criteria, the reliance on a single assessor without formal inter-rater reliability testing represents a limitation that may introduce diagnostic variability. Future studies should include multiple independent assessors with reliability checks to enhance diagnostic consistency. Second, although patients with concurrent psychotic disorders were excluded to enhance sample homogeneity, variations in the types of traumatic events experienced by participants and the presence of comorbid mood symptoms such as anxiety or depression may affect the generalizability of the findings. Meanwhile, the limited sample size in this study may pose inherent statistical challenges for high-dimensional, small-sample neuroimaging prediction research. Third, while LOOCV was used to control for overfitting, the lack of an independent test set or external sample validation limits the model’s stability and generalizability, necessitating further external validation in the future. Fourth, although machine learning successfully identified predictive functional connections mapped to large-scale brain networks, the precise anatomical localization and functional interpretation of these connections remain insufficiently detailed, constraining their potential utility as neural biomarkers. Fifth, while individualized analysis improves predictive accuracy at the subject level, it may weaken the generalizability of group-level inferences. By definition, individualized approaches prioritize subject-level variability, which can reduce the ability to draw broad conclusions applicable to larger cohorts or to identify universal neurobiological signatures of PTSD. Additionally, the current predictive targets primarily rely on clinician-rated subjective scales, future studies should incorporate objective behavioral or physiological measures to enhance the reliability and precision of predictions.

## Conclusion

This study primarily employed machine learning to investigate the predictive value of resting-state FC at both individual-specific and group-based atlas levels for clinical symptoms in PTSD patients. Our findings demonstrate that individual-specific ROI-based FC derived from ultra-high-field MRI plays a crucial role in predicting PTSD symptoms, whereas atlas-level FC diminished predictive performance. The predictive connections involved complicated functional brain networks, with DAN contributing most significantly. And the predictive effect was primarily linked to bidirectional alterations in between-network connectivity, with a net positive correlation observed on average. Although further validation is necessary, this exploratory study provides preliminary evidence suggesting that individual-specific ROI-based FC may hold potential for predicting PTSD symptomatology. This work advances the development of individualized neuroimaging biomarkers, with potential future applications in patient stratification, targeted neuromodulation, and objective treatment monitoring in PTSD.

## Supplementary Information

Below is the link to the electronic supplementary material.

Supplementary Material 1

## Data Availability

The data in the current study are available from the corresponding author on reasonable request.

## Abbreviations

PTSD: Post-traumatic stress disorder
FC: Functional connectivity
LOOCV: Leave-one-out cross-validation
PCL-5: Posttraumatic stress disorder checklist-5
ROI: Regions of interest
SVR: Support vector machine for regression
PFC: Prefrontal cortex
DMN: Default mode network
CEN: Central executive network
DAN: Dorsal attention network
SMN: Somatomotor network
SN: Salience network
VSL: Visual network
LMB: Limbic network
VAN: Ventral attention network
