# Supplementary material for: Individual-specific functional connectivity predicts clinical symptoms severity in patients with post-traumatic stress disorder
Source: BMC Psychiatry. 2026 Mar 12;26:331. doi: 10.1186/s12888-026-07969-3 (PMC13097695; doi:10.1186/s12888-026-07969-3)
Supplement: Supplementary file 1 — Supplementary Material 1 [file 12888_2026_7969_MOESM1_ESM.docx]

**Supplementary Methods**

**Permutation Test for Model Validity**

To evaluate whether the predictive performance of our SVR model was significantly above chance level, we conducted a full-pipeline permutation test (N = 1000). For each iteration, the labels were randomly shuffled while keeping the feature matrix unchanged, followed by a full LOOCV procedure including feature selection and SVR training. The correlation coefficient (Pearson's *r*) between predicted and permuted labels was computed for each permutation, generating a null distribution of *r* values.

The actual prediction performance (based on true labels) was then compared to this null distribution to compute a one-sided *p*-value, representing the probability of obtaining an equal or higher *r* under the null hypothesis. Both the atlas-level and individualized FC features were validated in the permutation test for model stabilities.

**Bootstrap-Based Confidence Interval Estimation**

To quantify the uncertainty of model predictions at the individual level, we employed a bootstrap-resampled LOOCV approach (N = 100). For each bootstrap iteration, we sampled *(N−1)* subjects with replacement, applied feature selection and SVR modeling, and recorded the predictions for the held-out subject.

After 100 repetitions, we obtained a distribution of predicted scores per subject, from which we computed the mean predicted value and 95% confidence intervals (CIs) using the empirical percentile method. The final scatter plots visualizing true vs. predicted values were overlaid with CI lines to enhance interpretability and applied to both atlas and individualized FC datasets.

**Sensitivity Analysis of Different Feature Selection Threshold**

To examine the robustness of model performance to the feature selection threshold, we conducted a sensitivity analysis using three different *p*-value thresholds (α = 0.001, 0.005, 0.01) during univariate feature selection. For each threshold, we performed LOOCV prediction and evaluated model performance using Pearson correlation (*r*) and mean absolute error (MAE) metrics.

This analysis allowed us to evaluate how the sparsity of selected features affected prediction accuracy. The procedure was visualized for both the atlas and individualized FC datasets, including separate plots for *r*-value and MAE across thresholds.

**Feature importance estimation**

To interpret the contribution of individual functional connections (FCs) in the predictive model, we implemented a permutation-based feature importance estimation procedure within each loop of the LOOCV framework. For non-linear SVR models (e.g., with Gaussian kernel), direct interpretation of feature weights is not straightforward. Therefore, we adopted a model-agnostic approach by quantifying the change in prediction performance when perturbing each selected feature. Specifically, for each left-out sample in the LOOCV, we first selected significant FC features from the training set using a predefined threshold. Then, for each selected feature, we permuted its values across the training samples (random shuffling) while keeping all other features unchanged. A new SVR model was retrained on this permuted dataset, and its prediction on the test sample was compared with the original prediction (before permutation). The absolute difference in predicted values was treated as the contribution score of that feature. Repeating this process across all samples and summing up the changes for each feature yielded an overall feature importance estimate.

This method ensures that the feature importance reflects the model's sensitivity to each FC feature, and avoids relying on kernel-specific weights, which are not interpretable in non-linear models.

**Regression of covariates**

Given the relatively modest sample size of our cohort, including multiple covariates in the regression model would increase the risk of overfitting, potentially compromising the generalizability and stability of our predictive model. To assess the influence of age, sex and head motion on symptom severity, we performed Pearson correlation analyses (for age and head motion) and Point-Biserial correlation analysis (for sex) between these demographic variables and PCL-5 scores in the PTSD patients.

**Supplementary Results**

**Permutation Test Analysis**As shown in Figure S1 and S2, the labels of both two models were randomly shuffled 1000 times, and the prediction procedure was repeated to obtain the null distribution of r-values under the hypothesis of no true association.

The individualized (indivi) model showed a strong prediction correlation (*r* = 0.5275, *p* = 0.0010), which lay in the extreme tail of the permutation distribution, yielding a significant p-value of *p* = 0.001 (Figure S1). This confirms that the individualized FC model captures a meaningful association beyond chance. In contrast, the atlas-based model failed to show statistically significant prediction performance (*r* = -0.0360, *p* = 0.3710), as the observed *r* fell within the central bulk of the permutation distribution (Figure S2).

**Bootstrap-augmented LOOCV analysis**

The bootstrap-augmented LOOCV analysis provided confidence intervals for each subject's predicted PCL-5 score. In the individualized model (Figure S3), the predicted values showed tight alignment with observed values (*r* = 0.5275), and narrow confidence intervals suggested high stability. In contrast, the atlas-based model (Figure S4) exhibited weaker prediction correlation (*r* = -0.0360), with wider confidence intervals, indicating less reliable predictions.

**Sensitivity Analysis across different α threshold**

For the atlas-based model, the prediction performance remained low and relatively stable across α levels, with r-values ranging from approximately -0.1 to 0.1 and MAEs slightly decreasing with more lenient thresholds.

In contrast, the indivi model showed higher sensitivity to the α threshold. The model achieved the best performance (r ≈ 0.5, MAE ≈ 7.8) when α = 0.005, while both more conservative and more liberal thresholds (α = 0.001 and 0.01) resulted in degraded performance.

**Correlation analysis of covariates**

No correlation was found between common confounding factors and PCL-5 scores (age: *r* = -0.0490, *p* = 0.7494, Figure S7a; sex: *r* = -0.0233, *p* = 0.8790; head motion: *r* = 0.2059, *p* = 0.1748, Figure S7b) , indicating that these factors did not substantially contribute to clinical symptom variation in our sample.

**Supplementary Figures**

**Figure S1. Permutation test for the individualized connectivity model (indivi).** The histogram shows the null distribution of Pearson correlation coefficients (r-values) obtained by 1000 label permutations under the leave-one-out cross-validation (LOOCV) framework. The red vertical line indicates the true model’s r-value computed using the original (unshuffled) data. The dashed line marks the chance level. The blue histogram bars indicate the null distribution of *r*-values derived from permuted labels and the true model's performance exceeds the permutation distribution, indicating statistical significance.

**Figure S2. Permutation test for the group-level atlas-based connectivity model (atlas).** Same as Figure S1, the permutation test was repeated 1000 times for the atlas-based model under the LOOCV framework. The red vertical line indicates the true model’s r-value computed using the original (unshuffled) data. The dashed line marks the chance level. The blue histogram bars indicate the null distribution of *r*-values derived from permuted labels and the red line indicates the *r*-value obtained from the true (unshuffled) data.


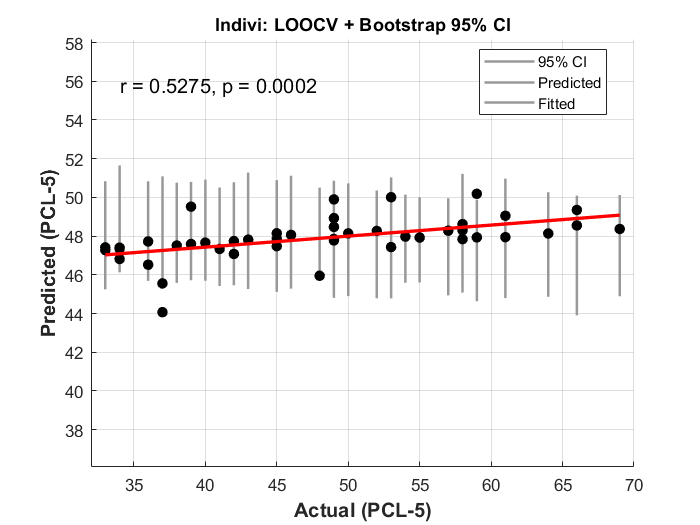


**Figure S3. Predictive performance of the indivi-based model with LOOCV and bootstrap-derived 95% confidence intervals (CI).** The scatter plot shows predicted versus observed PCL-5 scores using the indivi-based FC model under LOOCV. Each point represents one subject. Vertical bars represent 95% bootstrap confidence intervals of predicted values (100 bootstrap resamples). Annotated *r* and *p* values are computed from LOOCV predictions.

**Figure S4. Predictive performance of the atlas-based model with LOOCV and bootstrap-derived 95% confidence intervals (CI).** The scatter plot shows predicted versus observed PCL-5 scores using the atlas-based FC model under LOOCV. Each point represents one subject. Vertical bars represent 95% bootstrap confidence intervals of predicted values (100 bootstrap resamples). Annotated *r* and *p* values are computed from LOOCV predictions.


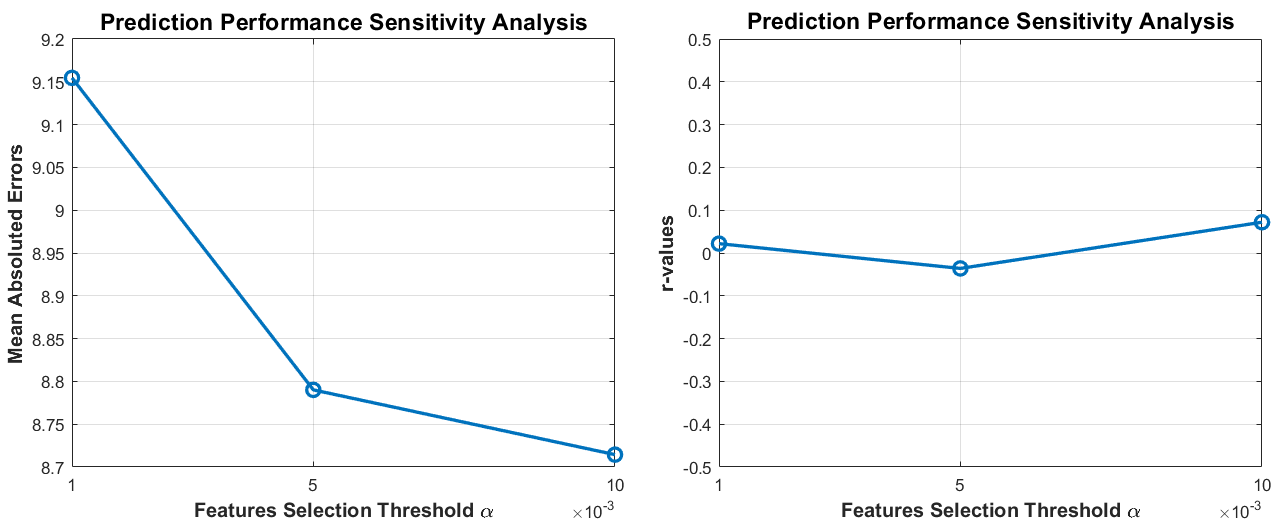


**Figure S5. Atlas-based model sensitivity analysis.** Sensitivity analysis of the atlas-based prediction model using different feature selection thresholds (α = 0.001, 0.005, 0.01). The left panel shows the mean absolute errors (MAE) for each α level, while the right panel shows the corresponding Pearson r-values.


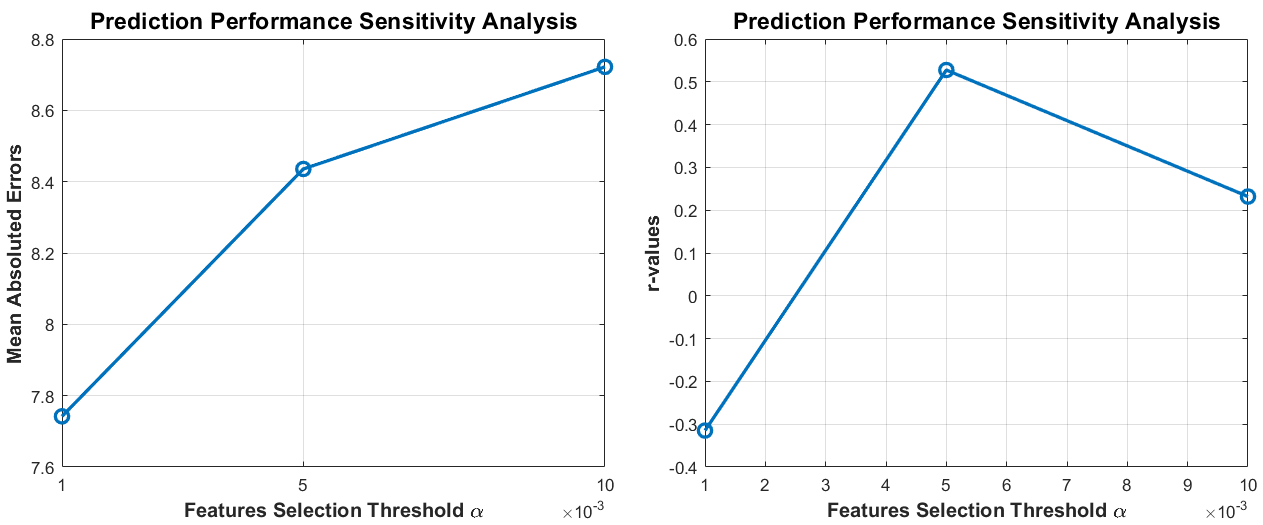


**Figure S6. Indivi model sensitivity analysis.** Sensitivity analysis of the individualized FC prediction model across three α levels (0.001, 0.005, 0.01). MAEs and r-values are plotted on the left and right panels, respectively.


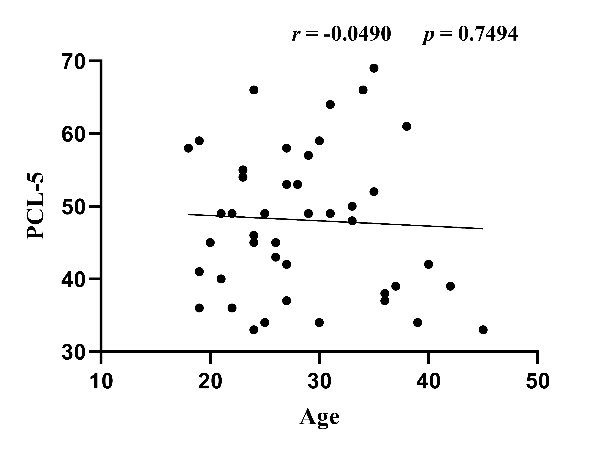

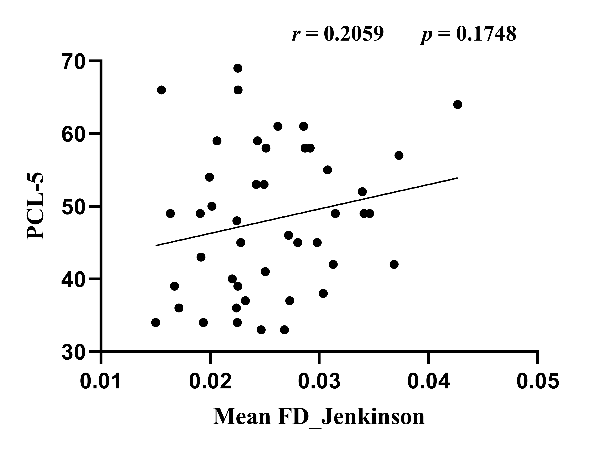


a b

**Figure S7. Pearson correlation analysis of covariates and actual PCL-5 scale scores.** a. Correlation analysis between age and actual PCL-5 scale scores; b. Correlation analysis between head motion parameter (FD) and actual PCL-5 scale scores.
